# HDAC3 Activity within the Nucleus Accumbens Regulates Cocaine-Induced Plasticity and Behavior in a Cell-Type-Specific Manner

**DOI:** 10.1523/JNEUROSCI.2829-20.2021

**Published:** 2021-03-31

**Authors:** R. R. Campbell, E. A. Kramár, L. Pham, J. H. Beardwood, A. S. Augustynski, A. J. López, O. S. Chitnis, G. Delima, J. Banihani, D. P. Matheos, M. A. Wood

**Affiliations:** ^1^Department of Neurobiology and Behavior, School of Biological Sciences, University of California, Irvine, California; ^2^University of California Irvine Center for Addiction Neuroscience, School of Biological Sciences, University of California, Irvine, California; ^3^Center for the Neurobiology of Learning and Memory, School of Biological Sciences, University of California, Irvine, California; ^4^Department of Pharmacology, Vanderbilt University School of Medicine, Nashville, Tennessee

**Keywords:** addiction, epigenetics, NAc

## Abstract

Epigenetic mechanisms regulate processes of neuroplasticity critical to cocaine-induced behaviors. This includes the Class I histone deacetylase (HDAC) HDAC3, known to act as a negative regulator of cocaine-associated memory formation within the nucleus accumbens (NAc). Despite this, it remains unknown how cocaine alters HDAC3-dependent mechanisms. Here, we profiled HDAC3 expression and activity in total NAc mouse tissue following cocaine exposure. Although chronic cocaine did not affect expression of *Hdac3* within the NAc, chronic cocaine did affect promoter-specific changes in HDAC3 and H4K8Ac occupancy. These changes in promoter occupancy correlated with cocaine-induced changes in expression of plasticity-related genes. To causally determine whether cocaine-induced plasticity is mediated by HDAC3's deacetylase activity, we overexpressed a deacetylase-dead HDAC3 point mutant (HDAC3-Y298H-v5) within the NAc of adult male mice. We found that disrupting HDAC3's enzymatic activity altered selective changes in gene expression and synaptic plasticity following cocaine exposure, despite having no effects on cocaine-induced behaviors. In further assessing HDAC3's role within the NAc, we observed that chronic cocaine increases *Hdac3* expression in *Drd1* but not *Drd2*-cells of the NAc. Moreover, we discovered that HDAC3 acts selectively within D1R cell-types to regulate cocaine-associated memory formation and cocaine-seeking. Overall, these results suggest that cocaine induces cell-type-specific changes in epigenetic mechanisms to promote plasticity important for driving cocaine-related behaviors.

**SIGNIFICANCE STATEMENT** Drugs of abuse alter molecular mechanisms throughout the reward circuitry that can lead to persistent drug-associated behaviors. Epigenetic regulators are critical drivers of drug-induced changes in gene expression. Here, we demonstrate that the activity of an epigenetic enzyme promotes neuroplasticity within the nucleus accumbens (NAc) critical to cocaine action. In addition, we demonstrate that these changes in epigenetic activity drive cocaine-seeking behaviors in a cell-type-specific manner. These findings are key in understanding and targeting cocaine's impact of neural circuitry and behavior.

## Introduction

Drugs of abuse alter gene expression and cellular activity within the reward system to promote drug-seeking behaviors. Many key epigenetic mechanisms that regulate gene expression and neuroplasticity are affected by drugs of abuse (Walker and Nestler, 2018; Campbell and Wood, 2019; López et al., 2020). More recently, cocaine-induced changes in plasticity are shown to occur within particular cell subtypes to drive changes in behavior (Lobo et al., 2010; Pascoli et al., 2011; Maze et al., 2014; Calipari et al., 2016; Roberts-Wolfe et al., 2018). However, it is unknown whether epigenetic mechanisms are responsible in initiating and maintaining cell-type-specific changes in plasticity that lead to persistent changes in behavior.

The Class I histone deacetylase (HDAC), HDAC3, is an epigenetic enzyme known to be a critical negative regulator of memory formation (McQuown and Wood, 2011; McQuown et al., 2011; Kwapis et al., 2017; Malvaez et al., 2018) and cocaine-associated changes in the nucleus accumbens (NAc; Malvaez et al., 2013; Rogge et al., 2013). Yet, it is still unclear how cocaine affects HDAC3-dependent mechanisms within the NAc. Previous work demonstrates that genetic deletion of *Hdac3* in the NAc alters histone acetylation and enhances cocaine-associated memory formation (Malvaez et al., 2013; Rogge et al., 2013). However, these manipulations disrupt HDAC3 protein-protein interactions, which are critical for memory formation (McQuown et al., 2011; Sun et al., 2013; Taniguchi et al., 2017; Penrod et al., 2018). Selective ablation of HDAC3 enzymatic function disrupts habitual learning and memory formation (Kwapis et al., 2017; Malvaez et al., 2018), yet it is unknown whether HDAC3 enzymatic activity is critical in cocaine response within the NAc.

The two major cell-types and medium spiny output neurons of the NAc (D1R-MSNs vs D2R-MSNs), have unique contributions to reward and motivated behaviors (Lobo et al., 2010). This is thought to occur, in part, because cocaine exerts unique cellular and molecular adaptations within D1R-MSNs versus D2R-MSNs (Jordi et al., 2013; Chandra et al., 2015; Calipari et al., 2016). However, the epigenetic mechanisms underlying cocaine-induced adaptations within these cell-types remain understudied. Moreover, HDAC3's role in this cell-type-specific regulation of cocaine-induced behaviors is not fully understood.

To address these key open questions, we characterized *Hdac3* expression and HDAC3 activity in the NAc following chronic cocaine exposure. Although cocaine did not alter *Hdac3* expression levels, cocaine selectively alters expression of downstream HDAC3-gene targets. To determine the functional contribution of HDAC3 in cocaine action, we overexpressed a deacetylase-dead HDAC3 point mutant (HDAC3-Y298H-v5) within the NAc of adult mice (Lahm et al., 2007). Disrupting HDAC3's activity altered target-specific changes in gene expression and synaptic plasticity in the NAc following cocaine exposure, but not behavioral responses to cocaine. To further understand HDAC3's role in the NAc, we next examined whether HDAC3 acts within a particular cell-type to drive cocaine-induced behaviors. We found that HDAC3 acts within primarily D1R-MSNs to affect cocaine-associated memory formation and cocaine-seeking. Together, these data suggest that HDAC3 is a key epigenetic regulator of cocaine-induced cell-type-specific plasticity and behavior.

## Materials and Methods

### 

#### Mice

C57BL/6 J mice, D1R- Cre and D2R- Cre mice were all single-housed and within 8–15 weeks old during behavioral testing. Drd1-Cre (EY262Gsat) and Drd2-Cre (ER44Gsat) mice were crossed with C57BL/6 J mice to breed hemizygous Drd1-Cre and Drd2-Cre mice for all experiments. Adult male mice were used for all global NAc HDAC3-Y298H-v5 experiments. Male and female mice were used for all cell-type-specific HDAC3-Y298H-v5 experiments. Mice were provided with food and water *ad libitum* for all experiments. Lights were maintained on a 12/12 h light/dark cycle, with all behavioral tests performed during the light portion of the cycle. All experiments were conducted according to National Institutes of Health *Guidelines for Animal Care and Use* and were approved by the Institutional Animal Care and Use Committee of the University of California, Irvine.

#### Drugs

Cocaine-HCl was purchased from Sigma-Aldrich and dissolved in saline (0.9% NaCl). Cocaine-HCl is expressed as the weight of the salt. For cocaine- CPP and cocaine-induced locomotion experiments, cocaine-HCl was dissolved and administered to a final dose of 5 or 10 mg/kg. Cocaine-HCl and saline were administered intraperitoneally. Animals were intraperitoneally injected with 20 mg/kg for both the electrophysiological recordings, chromatin immunoprecipitation (ChIP)-qPCR and RT-qPCR experiments. For RNAScope experiments, animals were intraperitoneally injected with 10 mg/kg cocaine. In intravenous self-administration sessions, mice had cocaine infusions at a dose of 0.5 mg/kg/infusion.

#### Adeno-associated virus (AAV) production

Wild-type HDAC3 was amplified from mouse hippocampal cDNA and cloned into a modified pAAV-IRES-hrGFP (Agilent), under control of the CMV promoter and β-globin intron. To create the point mutation, a single nucleotide substitution in exon 11 to direct production of a histidine residue in place of tyrosine at amino acid 298 was created. For the empty vector (EV) control, the HDAC3 coding sequence was not present, but all other elements remain. AAV was made by the Penn Vector Core (University of Pennsylvania) from the above-described plasmids and was serotyped with AAV 2.1. The final titer of AAV-HDAC3 (Y298H) was 6.48 × 1012 GC/ml and the final titer of AAV-EV was 1.35 × 1013 GC/ml.

For Cre-dependent vectors, products were subsequently cloned into a modified pAAV-hSyn-DIO-eGFP (Addgene #50 457, a generous gift from Bryan Roth) with the addition of β-globin intron. GFP element was removed from the original vector and replaced with a V5-tag, generating a fusion to HDAC3^Y398H^. This plasmid was then subsequently packaged into an AAV virus.

Viruses were packaged as described in López et al.(2019a). Briefly, HEK293 cells were transfected via standard calcium phosphate precipitation and grown in high-glucose-containing (4.5 g/l) DMEM (Invitrogen) supplemented with 10% fetal bovine serum (Life Technologies/Invitrogen), 100 units/ml penicillin and 100 µg/ml streptomycin at 37°C in a 5% humidified environment. Two hours before transfection, HEK293 cells were bathed in 5% fetal bovine serum in 25 ml IMDM (Invitrogen). Cells were transfected with: 12 ml H_2_O, 1.65 ml of 2.5 M CaCl_2_, plus AAV1 (30 μg), AAV2 (31.25 μg) and helper plasmid (125 μg), combined with target plasmid (62.5 μg) either rAAV-hSyn-DIO-V5-HDAC3^Y298H.^ 13 ml of 2× HEBS was vortexed into transfection buffer; 24 h following transfection, cells were bathed in fresh DMEM. Following 60–65 h, transfected HEK293 were harvested into PBS, pelleted, and resuspended in 150 mm NaCl/20 mm Tris. Cells were subsequently lysed in 10% NaDeoxycholate and 50 U/ml benzonase. Cells were frozen at −20°C for at least 24 h and virus was purified using Heparin columns. AAVs were concentrated using Amicon Ultra-4 concentrators. Viral titer was verified using qPCR. Briefly, AAVs were heat inactivated and nucleotide extracted with proteinase K in ABI buffer (500 mm KCL, 100 mm Tris, pH 8.0, and 50 mm MgCl), incubated at 50°C for 1 h and 95°C for 20 min.

#### Surgery

Mice were induced with 4% isoflurane in oxygen and maintained at 1.5–2.0% for the duration of surgery. Animals were injected with either AAV-HDAC3 (Y298H)-v5 or AAV-EV (Kwapis et al., 2017). 0.5 µl of virus was infused bilaterally into the NAc [anteroposterior (AP): +1.3 mm; mediolateral (ML): ±1.1 mm; dorsoventral (DV): −4.5 mm relative to bregma]. Immunofluorescence was used to confirm expression of HDAC3-Y298H-v5. Viruses were infused at a rate of 6 µl/hr by using a 30-gauge Neuros Hamilton syringe (product #65459-01) mounted to either a Harvard Apparatus Nanomite Syringe Pump (product #MA1 70-2217) or Leica Biosystems Nanoinjector Motorized f/Stereotaxics (product #39462901). All infusions used the Leica Microsystems Angle Two Stereotaxic System. All animals were allowed to recover for a minimum of two weeks days before handling.

#### Cocaine conditioned place preference (CCP)

Following intracranial viral infusions and two weeks recovery, CPP was performed as described in previous studies (Malvaez et al., 2011; White et al., 2016; Alaghband et al., 2018; López et al., 2019a). Briefly, all mice were handled for 2 min for three consecutive days before the experiment (days 1–3). Baseline preferences for three compartments in the CPP apparatus were assessed by placing the animals in the center compartment of the apparatus with free access to three distinct compartments for 15 min (day 4). Time spent in each compartment was recorded. Following this pretest, mice were conditioned over 4 d, alternating each day with either cocaine-HCl (5 or 10 mg/kg, i.p.; Sigma) or 0.9% saline (days 5–8); 24 h following the last conditioning session, postconditioning preference was tested in animals while they were in a drug-free state (day 9). On testing day, animals were allowed to freely explore all compartments of the CPP apparatus to assess preference for 15 min, established as the difference between time spent in the cocaine-paired chamber and the saline-paired chamber, in seconds. Time spent was tracked automatically from MPEG videos using EthoVision 3.1 software (Noldus Technology).

In the electrophysiology experiments, animals underwent handling and pretesting as described above. Following preconditioning testing, animals were injected with either cocaine-HCl (20 mg/kg) or 0.9% saline before being confined to one conditioning compartment for 30 min. Electrophysiological recordings were conducted 24 h following conditioning.

#### Cocaine-induced locomotion

This test examines the locomotor activating effects of cocaine in animals following experimenter-administered cocaine injections (White et al., 2016). Mice were handled for 2 min for 3 d (days 1–3) and were habituated to the activity apparatus (Plexiglas open field with sawdust bedding; base 16 cm × 32 cm) for 30 min/d for two consecutive days (days 4–5). Following intracranial viral infusions and two weeks recovery, mice were randomized into two different treatment groups (saline or cocaine) and locomotor activity was recorded for 30 min after an intraperitoneal injection of 10 mg/kg cocaine-HCl or 0.9% saline for 5 d (days 6–10). Locomotor activity (total distance traveled) was monitored and tracked automatically from MPEG videos using EthoVision 3.1 software (Noldus Technology).

#### Elevated plus maze (EPM)

The plus-maze was conducted by an experimenter blind to the experimental groups. The maze consists of two open arms (30 × 5 cm) and two closed arms (30 × 5 × 15 cm), that are connected by a central platform (5 × 5 cm). The maze was elevated 40 cm above the floor. During the test, mice were recorded for 5 min on the apparatus, with initially placing each mouse onto the central platform facing one of the open arms. Between subjects, the maze was cleaned with 70% ethanol. The percentage of time spent in the closed and open arms was scored using ANY-maze software.

#### Intravenous self-administration

First, mice were surgically catheterized: mice were anesthetized with an isoflurane (1–3%)/oxygen vapor mixture during surgery and implanted with intravenous catheters. The catheter tubing was passed subcutaneously into the jugular vein. Following surgery, animals recovered for ≥48 h before self-administration. Subjects were then permitted to acquire intravenous cocaine self-administration during 2 h daily sessions for 10 consecutive days. Cocaine was delivered through the intravenous catheter by a Razel syringe pump (Med Associates). Each session was performed using two retractable levers (one active, one inactive). Completion of the response criteria on the active lever resulted in the delivery of an intravenous cocaine infusion (0.03 ml infusion volume; FR1TO20s schedule on days 1–3, days 4–10 on FR2TO20s) at a dose of 0.5 mg/kg/infusion with a cue light presentation. Responses on the inactive lever were recorded but had no scheduled consequences. Catheters were flushed daily with physiological sterile saline solution (0.9% w/v) containing heparin (100 USP U/ml). Subjects and their data were removed from the study if the catheter integrity was compromised as determined by visual leakage or intravenous propofol assessment (propofol sodium, Patterson Vet). Behavioral responses were automatically recorded by Med Associates software.

#### Cocaine-seeking tests

Following 10 d of cocaine intravenous self-administration paradigm (IVSA) mice underwent 1 or 30 d of abstinence. Mice were subjected to a single 1-h IVSA session under extinction conditions, in which an active lever response resulted in a presentation of a cue but not drug delivery. Mice were killed immediately following the seeking session and NAc tissue was collected to confirm viral expression.

#### ChIP

ChIP was performed as described previously (Kwapis et al., 2017) based on the protocol from the Millipore ChIP kit. Tissue was cross-linked with 1% formaldehyde (Sigma), lysed and sonicated, and chromatin was immunoprecipitated overnight with 5 μl of anti- HDAC3 (Millipore), anti-H4K8AC (Millipore) or 5 μl of anti-mouse IgG (negative control, Millipore). The immunoprecipitate was collected using magnetic protein A beads (Millipore). After washing, chromatin was eluted from the beads and reverse cross-linked in the presence of proteinase K before column purification of DNA. Fos, Nr4a1 and Nr4a2 promoter enrichment in ChIP samples was measured by quantitative real-time PCR using the Roche 480 LightCycler and SYBR green. Primer sequences for the promoters, designed by the Primer three program are listed below; 5 μl of input, anti-HDAC3 IgG, or anti-mouse IgG immunoprecipitate were examined in duplicate. To normalize ChIP-qPCR data, we used the percent input method. The input sample was adjusted to 100% and both the IP and IgG samples were calculated as a percent of this input using the formula: 100*AE^(adjusted input – Ct (IP)). An in-plate standard curve determined amplification efficiency (AE; see Table 1).

**Table 1.** Primers for ChIP-qPCR

Promoter	Forward primer	Reverse primer
Fos	TTCTCTGTTCCGCTCATGACGT	CTTCTCAGTTGCTAGCTGCAATCG
Nr4a1	Gatagaggggtgggctgaag	aaaagagctcagtccgacga
Nr4a2	TGAAGTCCGTGGTGATGCTA	CGGGACAACTGTCTCCACTT
Nr4a3	GAGGGAGGAGGAGGGTGACGTA	CATAGAGTGCCTGGAATGCGAGA

#### Quantitative RT-qPCR

RT-qPCR was performed as described previously (Kwapis et al., 2017; López et al., 2019b). One-millimeter punches were collected from NAc in a 500 M slice of tissue. RNA was isolated from punches using an RNeasy Minikit (QIAGEN) and cDNA was created using the Transcriptor First Strand cDNA Synthesis kit (Roche Applied Science). The following primers were used, designed using the Roche Universal Probe Library (see Table 2).

**Table 2.** Primer for RT-qPCR

Gene	Forward primer	Reverse primer	Roche probe #
Hprt5	TGCTCGAGATGTCTGAAGG	ATCACATTGTGGCCCCTCTGT	–
Fos	ggggcaaagtagagcagcta	agctccctcctccgattc	46
Nr4a1	agcttgggtgttgatgttcc	aatgcgattctgcagctctt	93
Nr4a2	ttgcagaatatgaacatcgaca	gttccttgagcccgtgtct	2
Nr4a3	gtgtcgggatggttaaggaa	gagggctcctgttgtagtgg	91
Per1	tgtccgtcaccagtcagtgt	ccaggcaggtcttccatc	22
GriA1	agggatcgacatccagagag	tgcacatttcctgtcaaacc	62
GriA2	gcaaacagaaattgcttatgga	agtccacattttatcaaacactgc	106
Hdac3	ttcaacgtgggtgatgactg	ttagctgtgttgctccttgc	32
Hdac4	gcacagttgcatgaacatatca	Ctccagtttccgctggtg	17
Hdac5	gcatgaactctcccaacgag	tctgggttgatactgcctctc	20

Hprt5 probes were conjugated to LightCycler Yellow 555 to allow for multiplexing in the Roche LightCycle 480 II machine (Roche Applied Sciences). All values were normalized to Hprt5 expression levels and each group was compared with a saline EV-Control to normalize any gene induced nonspecifically by transportation or injection stress. Analyses and statistics were performed using the Roche proprietary algorithms and REST 2009 software based on the Pfaffl method (Pfaffl, 2001, 2002).

#### Immunofluorescence

Following behavioral testing, animals were killed and brain tissue was flash-frozen in isopentane and collected for immunohistochemistry (IHC). Twenty-micrometer coronal sections were collected using a Leica CM 1850 cryostat at −20°C and mounted on slides. Slices were fixed in 4% PFA for 10 min, washed in 0.1 m PBS and permeated in 0.1% Triton X-100 in 0.1 m PBS. Slices were then blocked in blocking serum (8% NGS, 0.3% Triton X-100, in PBS; 1 h) and incubated at 4°C overnight in primary solution (2% NGS, 0.3% Triton X-100; anti-v5: 1:1000, Abcam). The slices were then incubated in secondary solution (2% NGS, 0.3% Triton X-100; Alexa Fluor goat anti-rabbit 488). Lastly, tissue was incubated for 15 min in a DAPI solution (1:10 000, Invitrogen). Slides were coverslipped using VectaShield Antifade mounting medium (Vector Laboratories).

The tissue was imaged by using Olympus Slide Scanner VSBX61. Fluorescence was quantified by using ImageJ. Briefly, background signal was collected from a soma-free region and subtracted from NAc signal. All values were normalized to v5-containing tissue.

#### Slice preparation and recording

Parasagittal slices containing the NAc core were prepared from WT mice infused with either HDAC3-Y298H-v5 or EV (approximately two months of age). Following isoflurane anesthesia, mice were decapitated and the brain was quickly removed and submerged in ice-cold, oxygenated dissection medium containing the following: 124 mm NaCl, 3 mm KCl, 1.25 mm KH_2_PO_4_, 5 mm MgSO_4_, 2.5 mm CaCl_2_, 26 mm NaHCO_3_, and 10 mm glucose. Following removal of the cerebellum and lateral aspects of both hemispheres, parasagittal slices (320 μm) were cut from the blocked brain using a FHC vibrating tissue slicer (Model:OTS-5000). The tissue was then transferred to an interface recording chamber containing preheated artificial cerebrospinal fluid (aCSF) of the following composition: 124 mm NaCl, 3 mm KCl, 1.25 mm KH_2_PO_4_, 1.5 mm MgSO_4_, 2.5 mm CaCl_2_, 26 mm NaHCO_3_, 10 mm glucose, and 10 μm picrotoxin to reduce feedforward inhibition. Slices were continuously perfused with this solution at a rate of 1.0–1.5 ml min^−1^, while the surface of the slices were exposed to warm, humidified 95% O_2_/5% CO_2_ at 31 ± 1°C. Recordings began following at least 1.5 h of incubation.

Stimulation of glutamatergic afferent fibres within the NAc was achieved by placing a bipolar stainless steel stimulation electrode (25 μm in diameter, FHC) just below the anterior commissure. Activation of field (f)EPSPs were recorded using a glass pipette (2–3 MΩ) positioned caudal or caudal–ventral to the stimulation electrode. Thus, correct placement of electrodes within the NAc was confirmed by visual inspection of the slice and comparison with mouse brain atlas (Paxinos and Watson; 0.84–1.08 lateral to midline). Two parasagittal slices/hemisphere containing a large portion of the NAc core were obtained for each animal. Pulses were administered at 0.05 Hz using a current that elicited a 30–40% maximal response. Measurements of fEPSP slope (measured at 10–90% fall of the slope) were recorded during a minimum 20-min stable baseline period at which time long-term potentiation (LTP) was induced by delivering three to five trains (intertrain interval of 1 min), each train containing three “theta” bursts, with each burst consisting of four pulses at 100 Hz and the bursts themselves separated by 200 ms (TBS). The stimulation intensity was not increased during the delivery of TBS. Data were collected and digitized by NAC 2.0 Neurodata Acquisition System (Theta Burst Corp.) and stored on a disk.

#### *In situ* hybridization

We performed RNAscope ISH for *Hdac3*, *Drd1*, and *Drd2* mRNA. 60 min after the last injection, we briefly anesthetized mice with pentobarbital (50 mg/kg, i.p.), perfused mice with 1× PBS, and extracted whole-brain tissue. Brains were then incubated in 4% PFA for 24 h, and 30% sucrose solution for at least 48 h. Brains were then flash frozen in isopentane and stored at −80°C until use. NAc coronal sections (35 μm) were mounted directly onto Superfrost Plus slides (Fisher Scientific). We used an RNAscope Multiplex Fluorescent Reagent Kit II (Advanced Cell Diagnostics) and performed the ISH assay according to the user manual for fixed-frozen tissue. Each RNAscope target probe used contains a mixture of 20 ZZ oligonucleotide probes that are bound to the target RNA, as follows: Hdac3-C1 probe, Drd1-C2 probe and Drd2-C3 probe. Slides were incubated in a 1:10,000 DAPI solution for 15 min and washed with 1× PBS two times before coverslipping. Immediately following last washes, slides were coverslipped with a VECTASHIELD fluorescent mounting medium (H-1400, Vector Laboratories); 60× NAc fluorescent images were captured using a confocal microscope (Leica SP8).

For analysis, number of Hdac3 puncta in Drd1 versus Drd2 cells were counted using Imaris software. An average was calculated (total number of Hdac3 puncta detected in Drd1 cells or Drd2 cells/number of Drd1 or Drd2 cells analyzed) in each slice per animal (one to three slices). We then normalized cocaine and saline averages to saline averages to determine how cocaine altered Hdac3 colocalization within each cell-type.

#### Experimental design and statistical tests

Graphpad Prism 7 was used. All data are expressed as mean ± SEM. For all RT-qPCR, ChIP-qPCR, IHC either a two-tailed Student's *t* test or Mann–Whitney was run. Data in figures on LTP were normalized to the last 10 min of baseline. The LTP experiment and conventional measures of baseline synaptic transmission including paired-pulse facilitation and input/output (I/O) curves were analyzed using a two-way repeated measure analysis of variance. For CPP and EPM, repeated-measures (RM) two-way ANOVA's with *post hoc* Sidak's tests were conducted. Locomotion within CPP was assessed with two-tailed *t* tests. RM three-way ANOVA's were conducted for cocaine-induced locomotion data. RNAScope data were analyzed with a two-tailed *t* test and two-way ANOVA test. For cocaine IVSA, RM two-way ANOVAs and unpaired *t* test were conducted. Significance was set at *p* < 0.05 for all tests.

## Results

### Chronic cocaine alters activity HDAC3, but not HDAC3 expression, in the NAc to drive changes in plasticity-related gene expression

**Figure 1.**
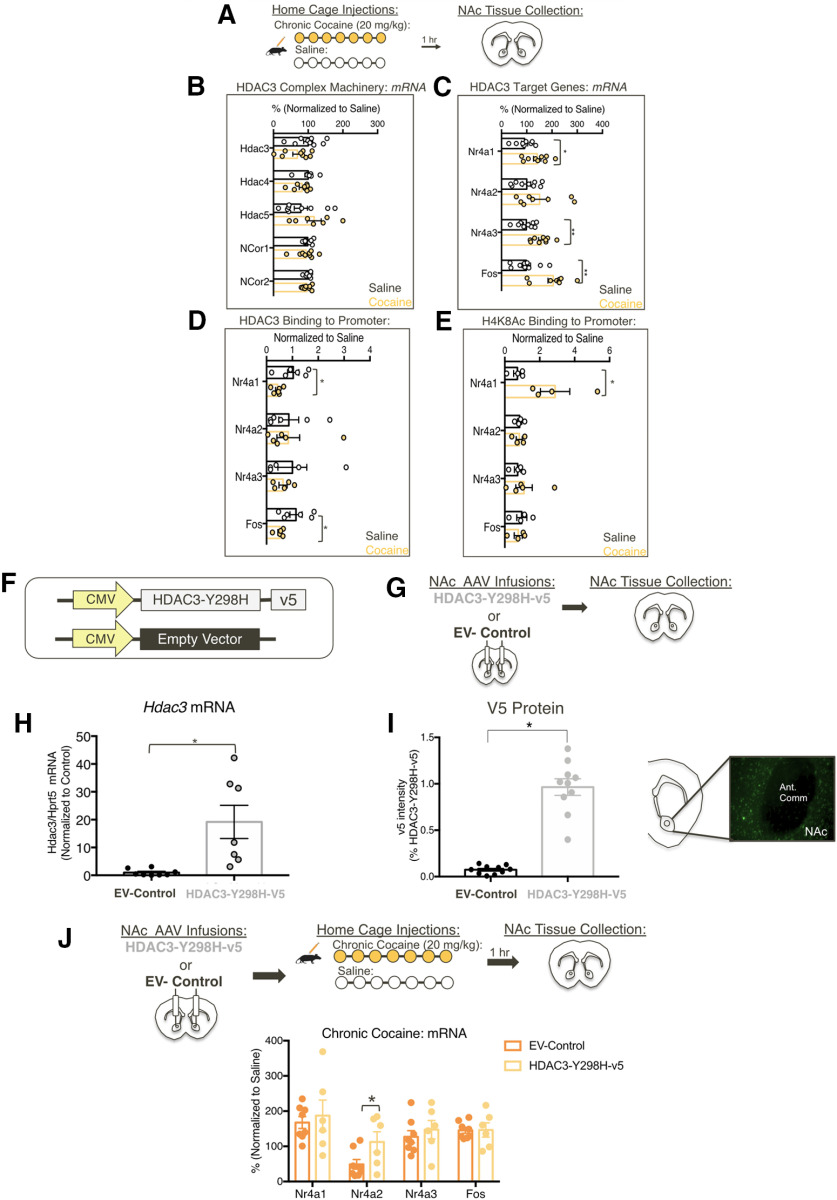
Cocaine alters the activity of HDAC3 to promote changes in gene expression in the NAc. ***A***, Adult male mice were intraperitoneally injected for 7 d with either cocaine (20 mg/kg) or saline, and tissue was collected 1 h following the last injection for either ChIP-qPCR or RT-qPCR. ***B***, Cocaine does not alter expression of Hdac3-related genes including *Hdac3*, *Hdac4*, *Hdac5, NCor1*, and *NCor2*. ***C***, Chronic cocaine injections alters mRNA levels of Nr4a1, Nr4a3, and *Fos*, but not Nr4a2 in the NAc. ***D***, ***E***, Chronic cocaine reduces HDAC3 binding and increases H4K8Ac binding to the promoter of Nr4a1, reduces HDAC3 binding to the promoter of Fos, but no changes were found on promoters of Nr4a2 or Nr4a3. HDAC3's deacetylase activity promotes cocaine-induced changes in *Nr4a2* expression in the NAc. ***F***, ***G***, Adult male mice were infused with AAV containing either deacetylase dead HDAC3 point mutant (HDAC3-Y298H-v5) or EV-Control. ***H***, ***I***, Overexpression of HDAC3-Y298H-v5 was confirmed by qPCR and immunofluorescence. ***E***, Mice infused with AAV containing either deacetylase dead HDAC3 point mutant (HDAC3-Y298H-v5) or EV-Control were exposed with either chronic cocaine or saline home cage injections; 1 h following the last injection, animals were killed and tissue was collected for RT-qPCR. ***J***, Disruption of HDAC3 activity enhances cocaine-induced changes in expression of *Nr4a2* mRNA following cocaine exposure. For RT-qPCR, samples were normalized to HPRT5 and EV-Control saline samples; *p < 0.05, **p < 0.01, ***p < 0.001.

We first examined how chronic cocaine exposure affects expression of HDAC3-regulated genes (McQuown et al., 2011; Rogge et al., 2013; Kwapis et al., 2017; López et al., 2019a) within the NAc using RT-qPCR (Fig. 1*A*). We found that *Hdac3* expression and other genes that comprise the HDAC3 complex, were unaffected by cocaine exposure (*Hdac3*: *t*_(16)_ = 1.708, *p* = 0.1070; *Hdac4*: *t*_(14)_ = 1.483, *p* = 1.603; *Hdac5*: *t*_(14)_ = 1.371, *p* = 0.1920; *NCor1*: *t*_(16)_ = 0.4492, *p* = 0.6593; *NCor2*: *t*_(14)_ = 0.7301, *p* = 0.4774; Fig. 1*B*). However, cocaine increases the expression of HDAC3-target genes *Nr4a1*, *Nr4a3* (Guez-Barber et al., 2011) and *Fos* (Guez-Barber et al., 2011; Larson et al., 2010; Kumar et al., 2005; Nr4a1: *t*_(15)_ = 2.546, *p < 0.05; Nr4a3: *t*_(16)_ = 3.689, *p < 0.05; *Fos*: *t*_(16)_ = 3.97, *p < 0.05; *Nr4a2*: *t*_(15)_ = 1.488, *p* = 0.1574; Fig. 1*C*). These findings suggest that cocaine does not alter the expression of HDAC3-related machinery (HDAC3-5, NCoR1/2), but does affect expression of downstream HDAC3-target genes in the NAc.

The above findings led to the hypothesis that chronic cocaine alters HDAC3 activity to promote plasticity in the NAc. Thus, ChIP-qPCR was used to examine whether enrichment of H4K8Ac and HDAC3 at the promoters of Nr4a1, Nr4a2, Nr4a3, and Fos changed following chronic cocaine (Li et al., 2000; McQuown et al., 2011; Kwapis et al., 2017; Malvaez et al., 2018). Chronic cocaine decreased HDAC3 occupancy at the promoters of both Nr4a1 and Fos. However, no changes in HDAC3 occupancy were observed on Nr4a2 or Nr4a3 promoters (Nr4a1: HDAC3 IP: *t*_(10)_ = 2.648, *p* = 0.0244; Fos: HDAC3 IP: *t*_(9)_ = 2.323, *p* = 0.0453; Nr4a2: HDAC3 IP: *t*_(10)_ = 0.03,444, *p* = 0.9732; Nr4a3: HDAC3 IP: *t*_(10)_ = 0.03,095, *p* = 0.9758; Fig. 1*D*). Elevated H4K8Ac levels were found only on the Nr4a1 promoter (Nr4a1: H4K8Ac IP: *t*_(6)_ = 2.491, *p* = 0.0471; Fos: H4K8Ac IP: *t*_(7)_ = 0.656, *p* = 0.5328; Nr4a2: H4K8Ac IP: *t*_(6)_ =0.1055, *p* = 0.09194; Nr4a3: H4K8Ac IP: *t*_(8)_ = 1.043, *p* = 0.3274; Fig. 1*E*). This suggests that cocaine alters HDAC3 activity at target-specific sites to promote changes in gene expression, in part mediated by changes in H4K8Ac levels, within the NAc.

### Disrupting HDAC3 activity alters *Nr4a2* expression within the NAc following cocaine exposure

We hypothesized that HDAC3's deacetylase activity is a key function in regulating cocaine-induced processes within the NAc. To test this, we used an AAV containing deacetylase-dead HDAC3 point mutant (HDAC3-Y298H-v5) that has been shown to affect HDAC3 deacetylase activity and memory dependent processes (Fig. 1*F*; Lahm et al., 2007; Kwapis et al., 2017; Malvaez et al., 2018). RT-qPCR (EV-Control: *n* = 8, median = 0.359; HDAC3-Y398H-v5: *n* = 7, median = 11.8; ***p < 0.001; Fig. 1*G*,*H*) and immunofluorescence (*t*_(18)_ = 9.898, *p* < 0.0001; Fig. 1*I*) confirmed that HDAC3-Y298H-v5 was expressed in the NAc. Together, these data indicate that viral overexpression of HDAC3-Y298H-v5 is sufficient to examine the importance of HDAC3's deacetylase activity in the NAc.

Given that HDACs negatively regulate cocaine-induced gene expression (Wang et al., 2010; Levine et al., 2011; Malvaez et al., 2013; Taniguchi et al., 2017), we examined whether disrupting HDAC3's deacetylase activity using HDAC3-Y298H-v5 affects cocaine-induced gene expression. We hypothesized that HDAC3-Y298H-v5 would enhance cocaine-induced gene expression of *Nr4a1/2/3* in the NAc. In contrast to our hypothesis, we found that only *Nr4a2* expression was affected by disrupting HDAC3 deacetylase activity following chronic cocaine in comparison to EV-Controls (*Nr4a1*: *t*_(12)_ = 0.4619, *p* = 0.6524; *Nr4a2*: *t*_(12)_ = 2.183, *p < 0.05; *Nr4a3*: *t*_(12)_ = 0.6746, *p* = 0.5127; *Fos*: *t*_(12)_ = 0.05628, *p* = 0.9569; Fig. 1*J*; Rogge et al., 2013; Kwapis et al., 2019). Together, these findings suggest that HDAC3's deacetylase activity regulates NAc gene expression in a target-specific manner.

### Disruption of HDAC3's deacetylase activity restores cocaine-induced changes in synaptic plasticity

**Figure 2.**
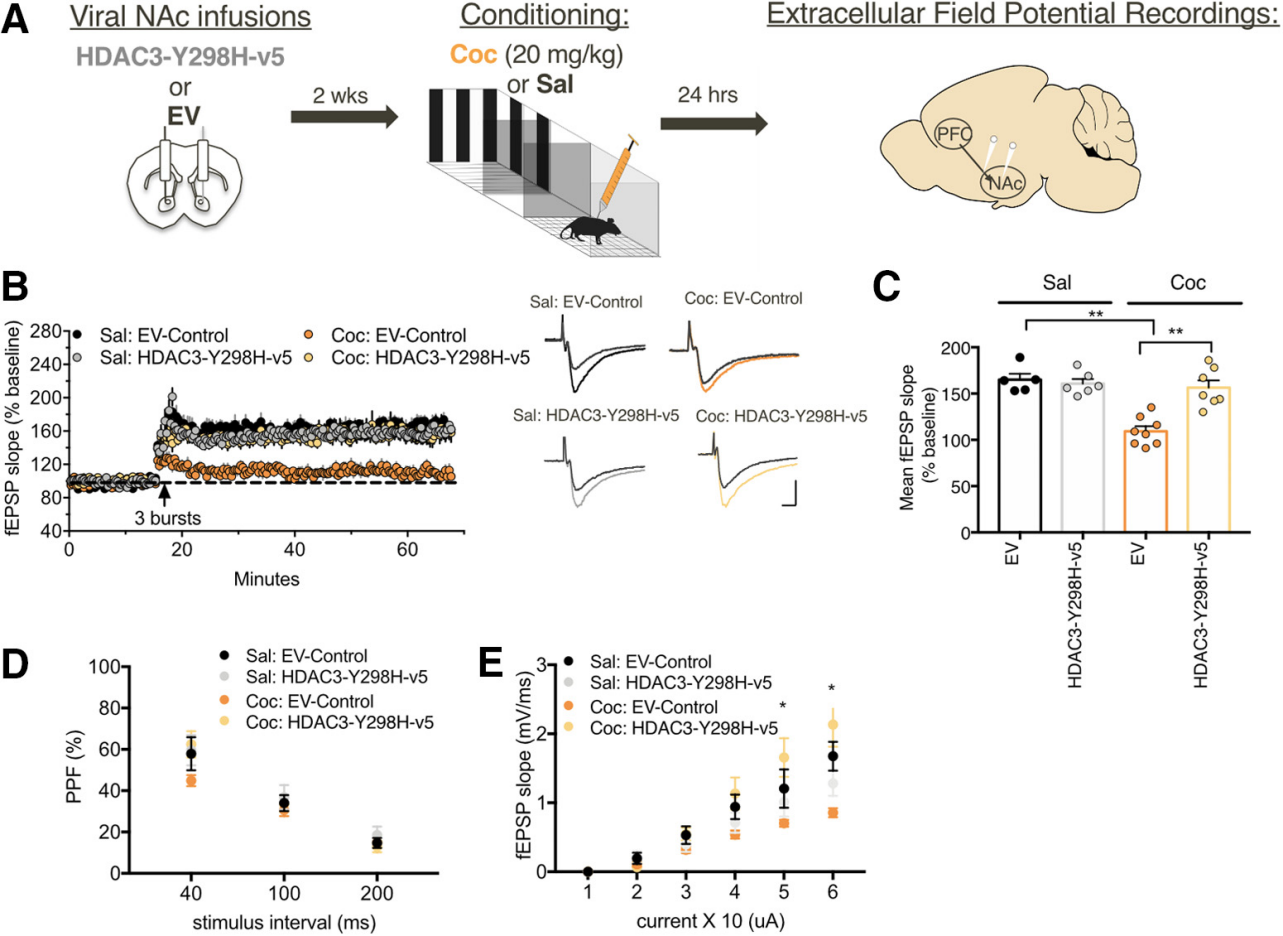
Disrupting HDAC3's activity reverses cocaine-induced synaptic plasticity in the NAc. ***A***, HDAC3-Y298H-v5 or EV-Control mice were injected with either cocaine (20 mg/kg) or saline before conditioning; 24 h following conditioning, extracellular field potential recordings were collected from the NAc following stimulation of glutamatergic afferents. ***B***, ***C***, NAc slices of EV-Control cocaine-conditioned mice had occluded LTP in comparison to EV-Control saline-conditioned mice. NAc slices from cocaine-conditioned animals infused with the HDAC3-Y298H-v5 virus had restored LTP in comparison to cocaine-conditioned EV-Controls. These effects were specific to cocaine-conditioned mice, as saline-conditioned mice exhibited similar potentiation regardless of virus. The effects of manipulating HDAC3 function on baseline neurotransmission were assessed using paired-pulse facilitation and with an I/O curve. ***D***, PPF was similar across groups, indicating that neither conditioning nor manipulation of HDAC3 activity had an effect on presynaptic release probability within the NAc. ***E***, HDAC3-Y298H-v5 had an effect on intrinsic membrane excitability in the NAc. Cocaine-conditioned slices had decreased NAc membrane excitability in comparison to EV-Controls, particularly at higher current injections. However, cocaine-conditioned HDAC3-Y298H-v5 slices exhibited higher levels of excitability in comparison to cocaine-conditioned EV-Controls at higher current injections. These collected data suggest that disrupting HDAC3 activity induces a physiological counteradaptation within NAc neurons; *p < 0.05, **p < 0.01, ***p < 0.001.

We next investigated whether HDAC3's deacetylase activity alters cocaine-induced changes in synaptic plasticity (Kourrich et al., 2007; Moussawi et al., 2009; Huang et al., 2013). Mice were infused with viruses containing either HDAC3-Y298H-v5 or EV and underwent either cocaine (20 mg/kg) or saline contextual conditioning; 24 h following conditioning, animals were killed and extracellular field potential recordings were collected from the NAc following stimulation of glutamatergic afferents (Fig. 2*A*; White et al., 2016). We predicted that disrupting HDAC3's activity would further depress LTP following theta-burst stimulation in cocaine-conditioned mice. Consistent with previous studies, NAc slices of EV-Control cocaine-conditioned mice occluded LTP in comparison to EV-Control saline-conditioned mice (Levine et al., 2011; Fig. 2*B*). However, NAc slices from HDAC3-Y298H-v5 cocaine-conditioned animals restored LTP similar to saline-conditioned EV-Controls (main effect of virus *F*_(1,22)_ = 10.91, *p* = 0.0032; main effect of cocaine *F*_(1,22)_ = 21.38, *p* = 0.0001; virus × cocaine interaction *F*_(1,22)_ = 15.77, *p* = 0.0006; Fig. 2*C*). HDAC3-Y298H-v5 effects were specific to cocaine-conditioned mice, as HDAC3-Y298H-v5 saline-conditioned mice exhibited similar potentiation as EV-Controls.

The effects of HDAC3-Y298H-v5 on baseline transmission in the NAc were next assessed using paired-pulse facilitation (PPF) to measure frequency facilitation and an I/O curve to detect changes in excitability (Fig. 2*D*). PPF was similar across groups, however HDAC3-Y298H-v5 had an effect on the I/O curve consistent with the LTP pattern (main effect of interval stimulus: *F*_(2,50)_ = 129.2, *p* < 0.001; no main effect of virus: *F*_(3,25)_ = 1.034, *p* = 0.3947; no interval stimulus × virus interaction: *F*_(6,50)_ = 1.446, *p* = 0.2163). Cocaine-conditioned slices had decreased NAc membrane excitability in comparison to EV-Controls, particularly at higher current settings (Zhang et al., 1998; Dong et al., 2006). However, cocaine-conditioned HDAC3-Y298H-v5 slices exhibited higher levels of excitability in comparison to cocaine-conditioned EV-Controls at the top of the curve (main effect of current: *F*_(5,125)_ = 118.5, *p* < 0.001; main effect of treatment: *F*_(3,25)_ = 3.973, *p* = 0.0192; current × treatment interaction: *F*_(25,125)_ = 6.299, *p* = 6.299; Fig. 2*D*). These collective data suggest that disrupting HDAC3 activity induces a physiological counteradaptation to cocaine, through altering membrane excitability, within the NAc.

### Global disruption of HDAC3 activity in the NAc does not affect cocaine-induced behaviors

**Figure 3.**
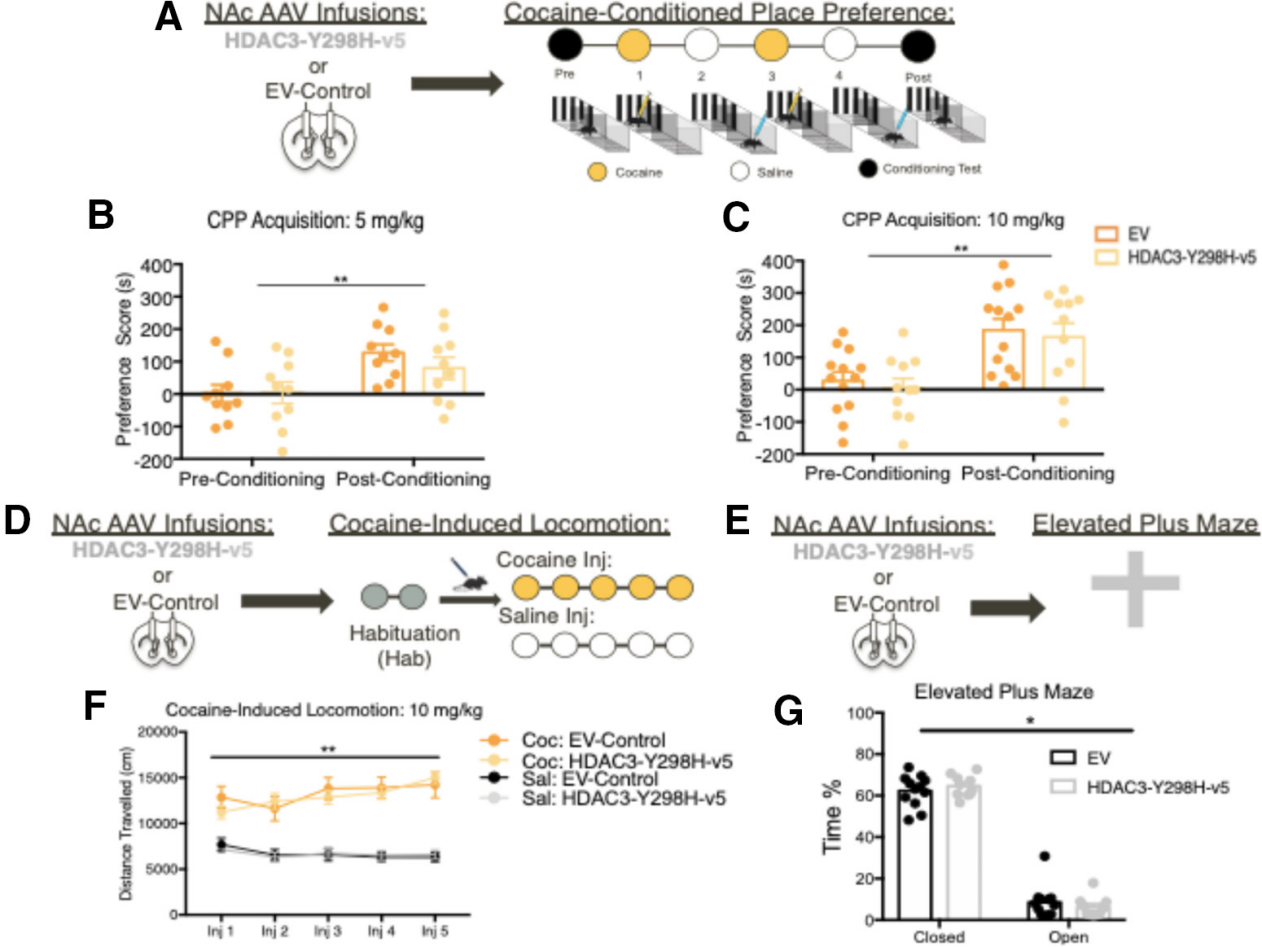
Global disruption of HDAC3's activity in the NAc does not alter behavioral responses to cocaine. ***A***, AAVs containing either HDAC3-Y298H-v5 or an EV-Control were infused into the NAc of adult male mice before cocaine CPP. ***B***, HDAC3-Y298H-v5 and EV-mice showed no initial preference for either chamber, and at a dose of 5 mg/kg, we found that HDAC3-Y298H-v5 had no effect on cocaine CPP. ***C***, Mice that were infused with either HDAC3-Y298H-v5 or EV-Control into the NAc underwent cocaine-CPP at a 10 mg/kg dose. Mice had no initial preference for either chamber before conditioning, and no differences were seen between HDAC3-Y298H-v5 and EV- controls following 10 mg/kg cocaine conditioning. ***D***, ***F***, Following AAV NAc infusions of HDAC3-Y298H-v5 or EV-Control, adult male mice received 5 d of injections of either cocaine or saline before being placed in an open chamber to track the total amount of distance traveled. Animals that received cocaine exhibited similar locomotor responses regardless of virus. ***E***, AAVs containing either HDAC3-Y298H-v5 or an EV-Control were infused into the NAc of adult male mice before EPM test. ***G***, HDAC3-Y398H-v5 and EV mice both spend significant amount of time in closed versus open arm in the EPM; *p < 0.05, **p < 0.01, ***p < 0.001.

**Figure 4.**
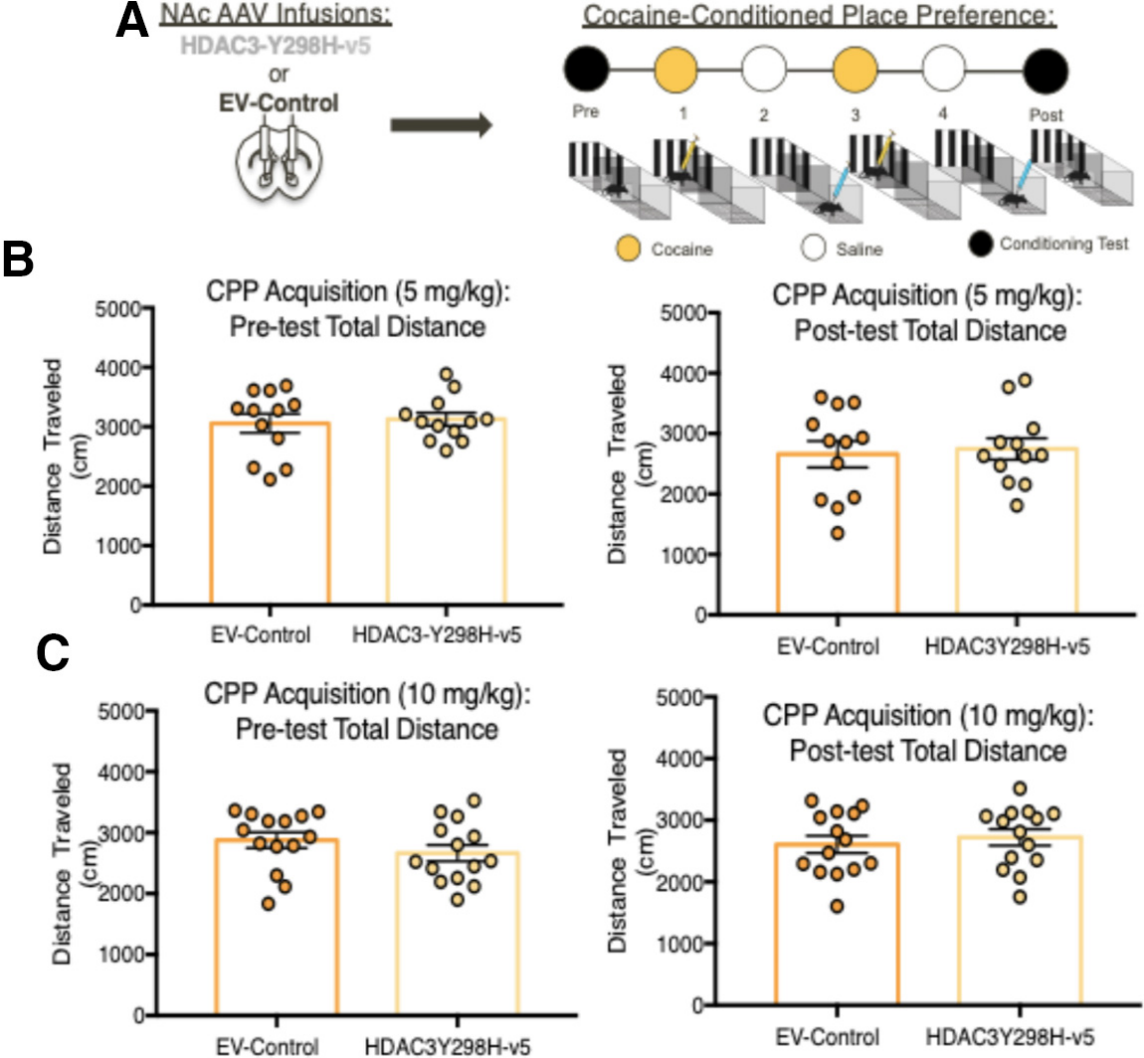
Disrupting HDAC3's activity has no effect on locomotor activity during pre/postconditioning test CPP days. ***A***, AAVs containing either HDAC3-Y298H-v5 or an EV-Control were infused into the NAc of adult male mice before cocaine CPP. ***B***, HDAC3-Y298H-v5 and EV-Control mice had no differences in total locomotion during the 5 mg/kg preconditioned testing day or the postconditioned testing day. ***C***, HDAC3-Y298H-v5 and EV-Control mice had no differences in total locomotion during the 10 mg/kg preconditioned testing day or the postconditioned testing day.

After seeing the molecular and cellular effects following disruption of HDAC3 activity within the NAc, we tested whether HDAC3-Y298H-v5 affects behavioral responses to cocaine. Surprisingly, HDAC3-Y298H-v5 had no effects on cocaine CPP (cocaine 5 mg/kg: main effect of conditioning *F*_(1,18)_ = 27.68, *p* < 0.0001; no main effect of virus *F*_(1,18)_ = 0.3477, *p* = 0.565; no conditioning × virus interaction *F*_(1,18)_ = 1.72, *p* = 0.2062; 10 mg/kg cocaine: main effect of conditioning *F*_(1,22)_ = 53.47, *p* < 0.0001; no main effect of virus *F*_(1,22)_ = 0.2522, *p* = 0.625; no conditioning × virus interaction *F*_(1,22)_ = 0.0001, *p* = 0.9906), cocaine-induced locomotion (three-way ANOVA: main effect of cocaine: *F*_(1,4)_ = 301.6, *p* < 0.0001; no main effect of virus: *F*_(1,4)_ = 3.312, *p* = 0.7323; no main effect on session: *F*_(4,4)_ = 1.01, *p* = 0.4035), or anxiety-like behaviors (EPM: two-way ANOVA RM: main effect of arm: *F*_(1,20)_ = 511.4, *p* < 0.0001; no main effect or virus: *F*_(1,20)_ = 0.003, *p* = 0.9568; no interaction: *F*_(1,20)_ = 0.861, *p* = 0.3645; Figs. 3*A–G*, 4*A–C*). Altogether, these data demonstrate that global disruption of HDAC3's deacetylase activity in the NAc does not affect cocaine-induced or baseline behaviors. From these findings, we hypothesized that our lack of behavioral effects may be because of the technical approach of globally disrupting HDAC3's deacetylase activity in the NAc.

### Chronic cocaine alters Hdac3 expression in Drd1 versus Drd2 cells of the NAc

**Figure 5.**
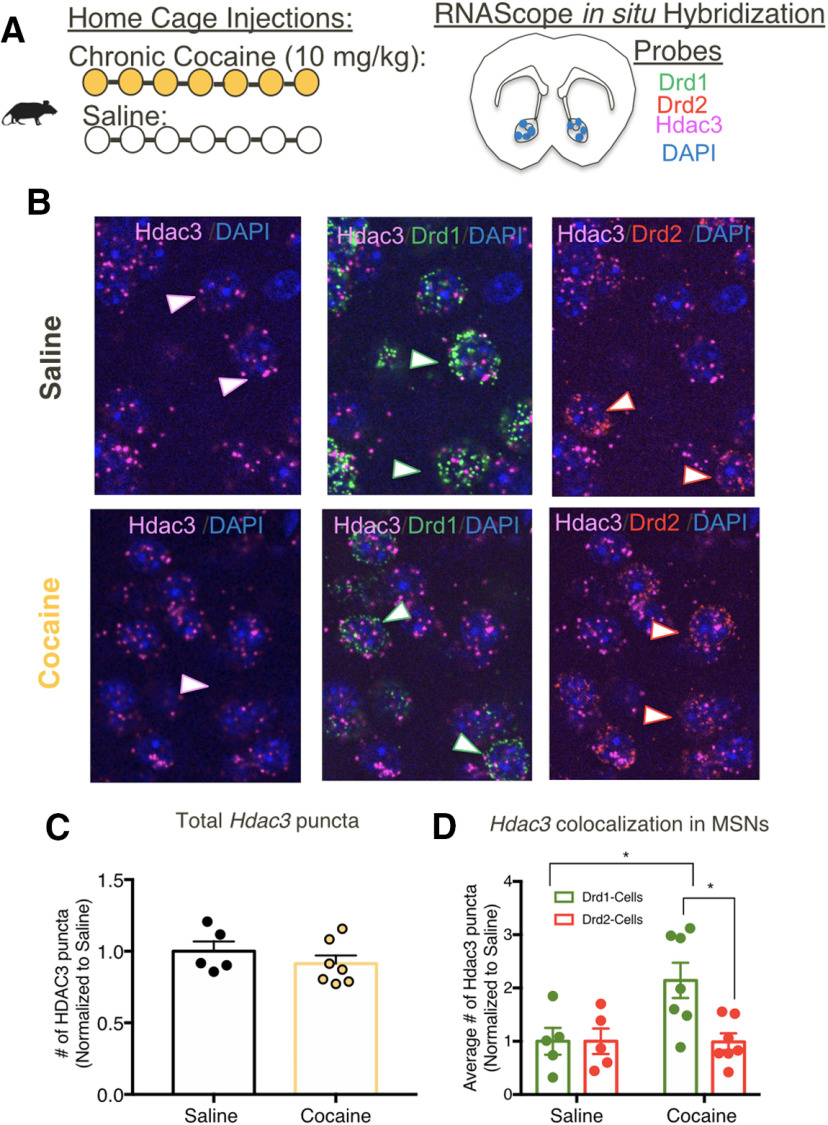
Cocaine alters Hdac3 expression within Drd1 versus Drd2 cells in the NAc. ***A***, Animals underwent chronic injections of either saline or cocaine and NAc tissue was collected to examine Hdac3 mRNA localization/expression in Drd1 versus Drd2 cells using *in situ* hybridization. ***B***, Triplex detection of *Drd1* (green), *Drd2* (red), and *Hdac3* (pink) mRNAs in NAc after chronic cocaine or saline injections. Representative images of selected cells. Images on the left (*Hdac3*+ *DAPI*) show merged channels for *Hdac3* (pink) and DAPI (blue) signals. Centered images show merged channels for Hdac3 (pink) *Drd1-DAPI* (green+blue) signals. Images on the right (*Hdac3*+*Drd2*) show the merged channels for *Hdac3* (pink) and *Drd2-DAPI* (red+blue) from the same brain sections. ***C***, Cocaine exposure does not alter Hdac3 expression. Counts of Hdac3 puncta detected in the NAc following cocaine or saline exposure. ***D***, Graphs indicating the average number of *Hdac3-*puncta coexpressed in *Drd1 (Hdac3*+*Drd1)* or *Drd2 (Hdac3*+*Drd2)* mRNA in the NAc. *Hdac3* puncta is increased in Drd1-containing cells following cocaine exposure; *p < 0.05, **p < 0.01, ***p < 0.001.

Although global HDAC3 manipulations showed no effects on cocaine-associated behaviors, we continued to investigate how HDAC3-related neuroplasticity within the NAc could ultimately affect behavior. Given that the two major cell-types (D1R-MSNs and D2R-MSNs) of the NAc have opposing roles in regulating cocaine-related behavior, we hypothesized that cocaine alters HDAC3 in a cell-type-specific manner within the NAc. We first tested whether *Hdac3* expression within *Drd1* versus *Drd2* expressing cells is altered by cocaine. Using RNAScope *in situ* hybridization, male and female mice underwent either 7 d of injections of cocaine (10 mg/kg) or saline (Fig. 5*A*,*B*). Consistent with our previous findings, cocaine does not alter *Hdac3* expression when quantifying within total NAc tissue (unpaired *t* test; *t*_(10)_ = 0.9785, *p* = 0.3509; Fig. 5*C*). However, when examining the effects of cocaine within Drd1 and Drd2 cells of the NAc, cocaine increased the average number of *Hdac3* puncta within Drd1-containing, but not Drd2-containing, cells (two-way ANOVA: interaction *F*_(1,10)_ = 7.491 *p* = 0.0209; no main effect of cocaine: *F*_(1,10)_ = 3.418 *p* = 0.0942; main effect of cells: *F*_(1,10)_ = 7.491 *p* = 0.0209; Sidak's test, *p* < 0.0034; Fig. 5*D*). This suggests that cocaine may differentially affect HDAC3 within the two major cell-types of the NAc to regulating cocaine-induced changes in plasticity.

### D1R-MSN-specific disruption of HDAC3's deacetylase activity in the NAc alters cocaine-associated memory formation and cocaine-seeking

**Figure 6.**
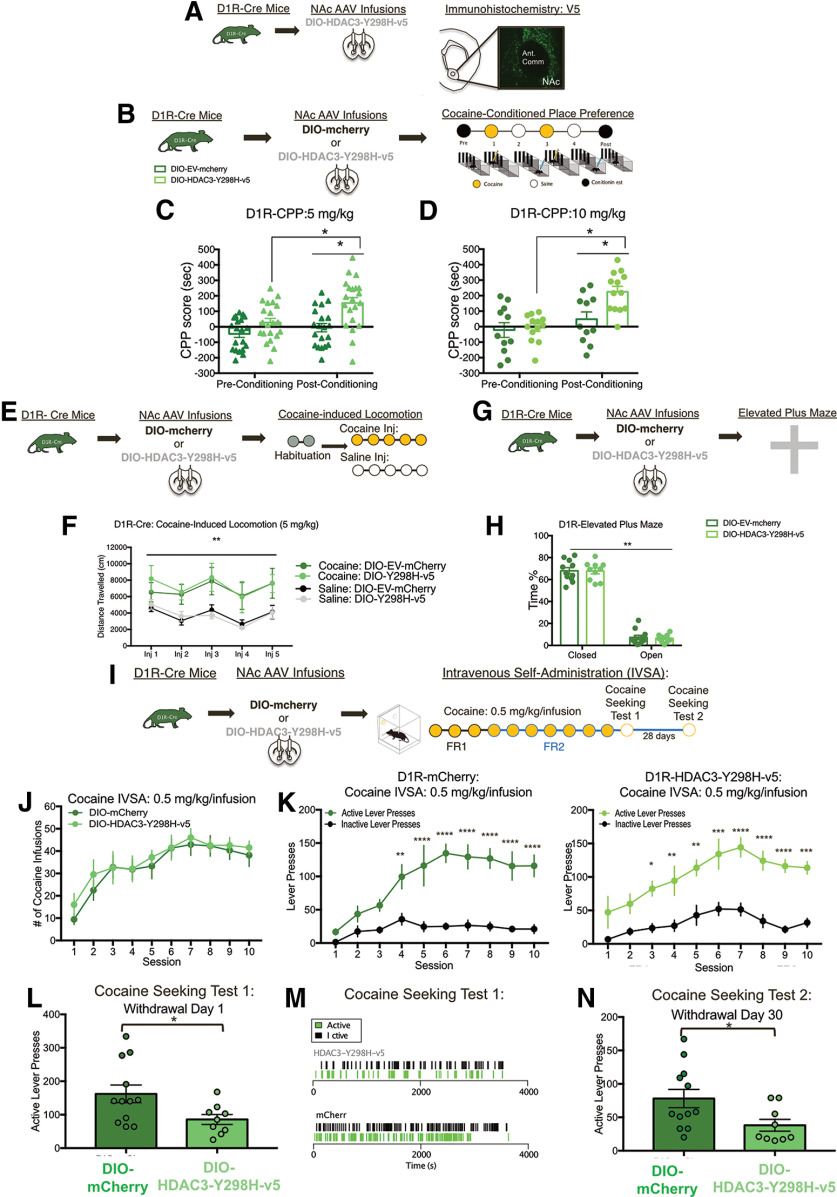
Disruption of HDAC3's deacetylase activity within D1R-MSNs alters cocaine-associated memory formation and cocaine seeking behaviors. ***A***, Representative image of V5 expression detected in the NAc of D1R-Cre mice that underwent AAV DIO-HDAC3-Y298H-v5 infusions. ***B***, AAVs containing either DIO-HDAC3-Y298H-v5 or an DIO-mCherry-control were infused into the NAc of adult D1R-Cre male or female mice before cocaine CPP. ***C***, HDAC3-Y298H-v5 and mCherry-mice showed no initial preference for either chamber, and at a dose of 5 mg/kg, we found that HDAC3-Y298H-v5 enhanced cocaine CPP. ***D***, Male and female D1R-Cre mice that were infused with either DIO-HDAC3-Y298H-v5 or DIO-mCherry-Control into the NAc underwent cocaine CPP at a 10 mg/kg dose. Mice had no initial preference for either chamber before conditioning; however, following 10 mg/kg cocaine conditioning, HDAC3-Y298H-v5 enhanced CPP acquisition. ***E***, AAVs containing either DIO-HDAC3-Y298H-v5 or DIO-mcherry were infused into the NAc of D1R-Cre adult male and female mice. Animals next underwent cocaine-induced locomotion test, where mice were subjected to 5-d intraperitoneal injections of either cocaine (5 mg/kg) or saline and placed in an open chamber to track distance traveled per session. ***F***, D1R-Cre mice that received cocaine exhibited significantly higher locomotor responses versus saline regardless of virus. ***G***, AAVs containing either DIO-HDAC3-Y298H-v5 or an DIO-EV-mcherry were infused into the NAc of adult D1R-Cre mice before EPM test. ***H***, D1R-males and females had spent significantly more time closed arms versus open arms, regardless of virus. ***I***, D1R-Cre mice were infused AAVs containing either DIO-mcherry or DIO-HDAC3-Y298H-v5 and two weeks following AAV infusions underwent catherization surgery. Following recovery, mice underwent cocaine IVSA conditions (FR1→FR2; 0.5 mg/kg/infusion) for 10 d and then underwent cocaine seeking tests 24 h and 30 d following last IVSA session. ***J***, Disrupting HDAC3 activity (overexpressing HDAC3-Y298H-v5) in NAc D1-MSNs had no effect on discrimination of active and inactive levers or (***K***) cocaine intake (FR1→FR2, 0.5 mg/kg/inf). ***L***, 24 h and 30 d after the last cocaine session, D1R-Y298H mice have decreased cocaine-seeking. ***M***, Representative recordings of D1-mcherry and D1R-HDAC3-Y298H-v5 mice illustrating lever pressing throughout the cocaine seeking test session. ***L***, 30 d after the last cocaine session, D1R-Y298H mice have decreased cocaine-seeking; *p < 0.05, **p < 0.01, ***p < 0.001.

**Figure 7.**
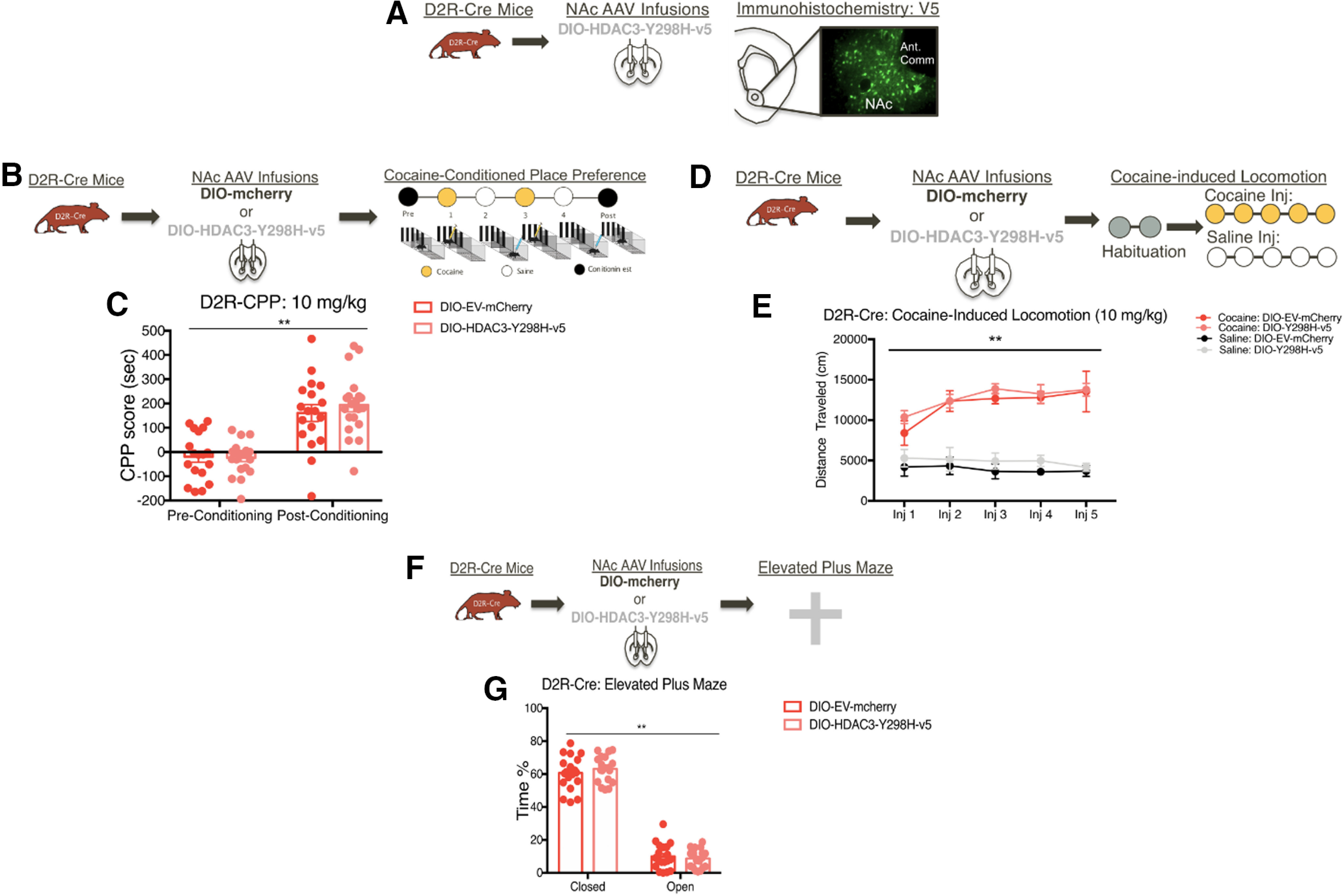
D2R-MSN-specific disruption of HDAC3's deacetylase activity in the NAc does not alter cocaine-induced behaviors. ***A***, Representative image of V5 expression detected in the NAc of D2R-Cre mice that underwent AAV DIO-HDAC3-Y298H-v5 infusions. ***B***, D2R-Cre mice that were infused with either DIO-HDAC3-Y298H-v5 or DIO-mCherry-Control into the NAc underwent cocaine-induced CPP at a 10 mg/kg dose. Following 10 mg/kg cocaine conditioning (***C***) HDAC3-Y298H-v5 did not affect CPP acquisition in males and females. ***D***, Following AAV NAc infusions of DIO-HDAC3-Y298H-v5 or DIO-mCherry-Control, adult male and female D2R-Cre mice received 5 d of injections of either cocaine or saline. ***E***, D2R-Cre mice exhibited similar cocaine-induced locomotion regardless of virus in comparison to saline locomotion. ***F***, AAVs containing either DIO-HDAC3-Y298H-v5 or an DIO-EV-mcherry were infused into the NAc of adult D2R-Cre mice before EPM test. ***G***, D1R-males and females had spent significantly more time closed arms versus open arms, regardless of virus; *p < 0.05, **p < 0.01, ***p < 0.001.

**Figure 8.**
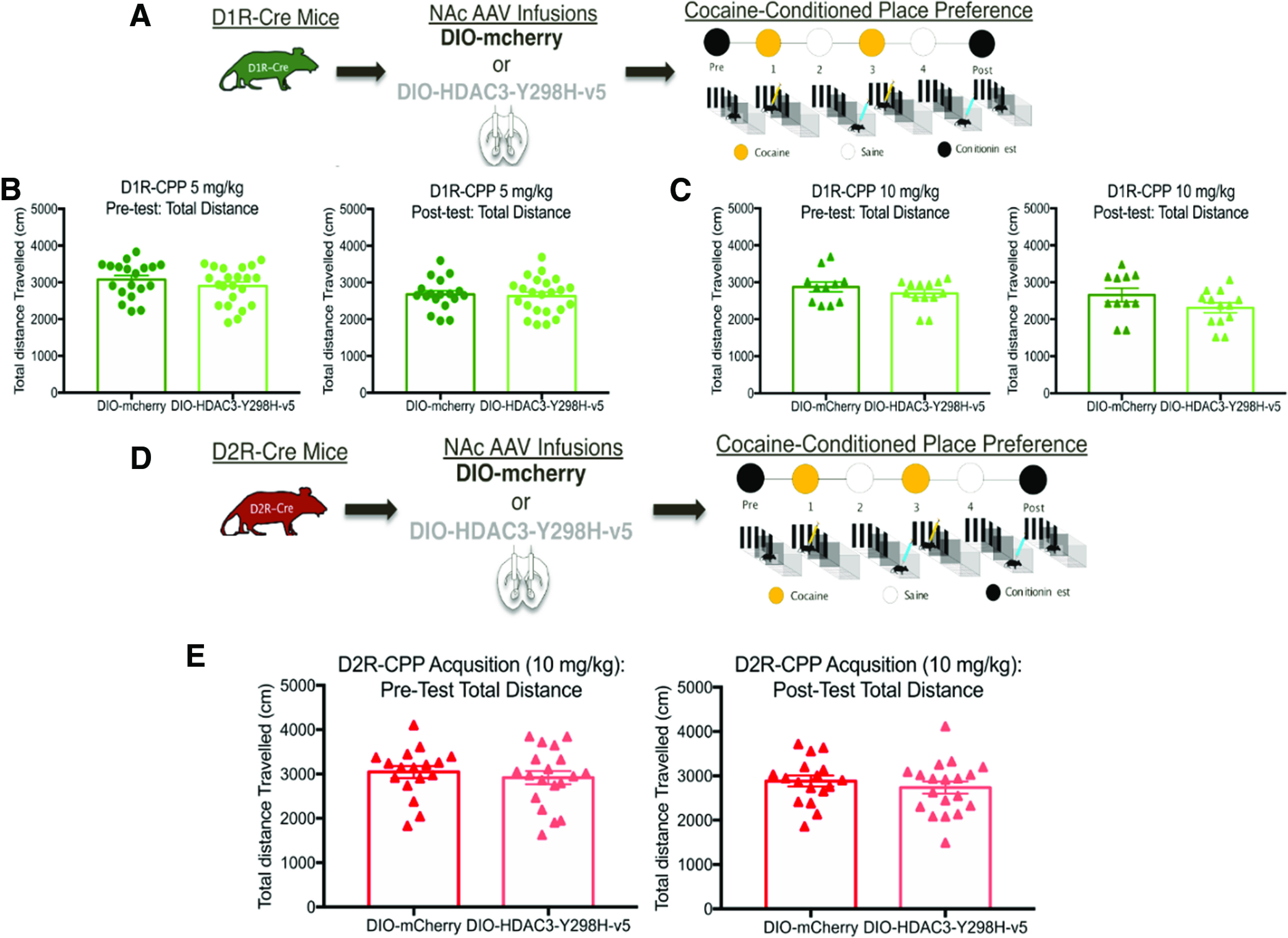
Disrupting HDAC3's activity in either D1R and D2R-cells has no effect on locomotion during pre/postconditioning test CPP days. ***A***, AAVs containing either DIO-HDAC3-Y298H-v5 or DIO-mCherry-Control were infused into the NAc of adult D1R-Cre mice before cocaine CPP. ***B***, In D1R-Cre male and female mice, DIO**-**HDAC3-Y298H-v5 and DIO-mCherry-Control had no significant effect on total distance during the 5 mg/kg preconditioned testing day or the postconditioned testing day. ***C***, DIO**-**HDAC3-Y298H-v5 and DIO-mCherry-Control mice had no significant effect on the amount of total distance traveled in the 10 mg/kg preconditioned testing day or the postconditioned testing day. ***D***, AAVs containing either DIO-HDAC3-Y298H-v5 or DIO-mCherry-Control were infused into the NAc of adult D1R-Cre mice before cocaine CPP. ***E***, DIO**-**HDAC3-Y298H-v5 and DIO-mCherry-Control male and female D2R-Cre mice had no significant effect on the amount of total distance traveled in the preconditioning testing or postconditioning testing in CPP 10 mg/kg.

To investigate the cell-type-specific role of HDAC3 within MSNs, we used Cre-dependent versions of HDAC3-Y298H-v5 (DIO-HDAC3-Y298H) combined with D1-Cre or D2-Cre transgenic mouse lines. Cre-dependent overexpression of HDAC3-Y298H-v5 in the NAc in both D1R-Cre and D2R-Cre mice was confirmed using IHC (Figs. 6*A*, 7*A*). Based on the known differential effects of cocaine within D1R-MSN-mediated versus D2R-MSN-mediated activity, we hypothesized that disrupting HDAC3's deacetylase activity within D1R-MSNs will enhance behavioral responses to cocaine, whereas HDAC3-Y298H-v5 within D2R-will impair cocaine-induced behaviors. HDAC3's role within these cell-types was first examined using cocaine CPP. Both D1R and D2R Cre male and female mice underwent AAV NAc infusions containing either a Cre-dependent HDAC3-Y298H-v5 or mCherry (Figs. 6*B*, 7*B*). Overexpressing HDAC3-Y2H98-v5 within D1R-Cre mice enhanced cocaine CPP (5 mg/kg: main effect of conditioning *F*_(1,18)_ = 11.79, *p* = 0.003; main effect of virus *F*_(1,18)_ = 10.57, *p* = 0.0044; no conditioning × virus interaction *F*_(1,18)_ = 1.394, *p* = 0.2532; 10 mg/kg: main effect of conditioning *F*_(1,16)_ = 10.3, *p* = 0.055; main effect of virus *F*_(1,16)_ = 5.704, *p* = 0.0296; conditioning × virus interaction *F*_(1,16)_ = 04.591, *p* = 0.0479; Fig. 4*C*,*D*). In contrast, HDAC3-Y298H-v5 within D2R-Cre mice had no effect on cocaine CPP (main effect of conditioning *F*_(1,36)_ = 125.4, *p* < 0.0001; no main effect of virus *F*_(1,36)_ = 0.1937, *p* = 0.6625; no conditioning × virus interaction *F*_(1,36)_ = 1.156, *p* = 0.2895; Fig. 7*A–C*). D1R-HDAC3-Y298H-v5 or D2R-HDAC3-Y298H-v5 had no effects on locomotor activity during CPP testing [D1R-Cre 5 mg/kg preconditioned testing day: unpaired *t* test *t*_(39)_ = 1.127, *p* = 0.2665; D1R-Cre 5 mg/kg postconditioned testing day: unpaired *t* test *t*_(39)_ = 0.305, *p* = 0.7631; D1R-10 mg/kg preconditioned testing day: unpaired *t* test *t*_(22)_ = 1.044, *p* = 0.308; D1R-10 mg/kg postconditioned testing day: unpaired *t* test *t*_(22)_ = 1.57, *p* = 1.307 (Fig. 8*A–C*); D2R-10 mg/kg preconditioning testing day: unpaired *t* test *t*_(34)_ = 0.6117, *p* = 0.5488; D2R-10 mg/kg postconditioning testing day: unpaired *t* test *t*_(34)_ = 0.7905, *p* = 0.4347 (Fig. 8*D*,*E*)], indicating these effects were unrelated to locomotor performance of the task. These data suggest that HDAC3's deacetylase activity within D1R-MSNs, but not D2R-MSNs, regulates cocaine CPP.

We further examined whether disrupting HDAC3's activity within D1R-MSNs versus D2R-MSNs alters cocaine-induced behaviors. Given our CPP data, we hypothesized that overexpressing HDAC3-Y298H-v5 within D1R-MSNs would enhance cocaine-induced locomotion. Thus, D1R-Cre mice were injected with a low dose of cocaine (5 mg/kg) to prevent ceiling effects. In contrast to our hypothesis, cocaine-treated D1R-Cre mice exhibited significantly higher locomotor responses versus saline regardless of virus (three-way ANOVA: main effect of cocaine: *F*_(1,4)_ = 43.13, *p* < 0.0001; no main effect of virus: *F*_(1,4)_ = 0.1575, *p* = 0.6923; no main effect on session: *F*_(4,4)_ = 2.152, *p* = 0.0800; Fig. 6*E*,*F*). In regards to D2R-MSNs, because D2R-MSN activity inhibits cocaine behavioral responses (Lobo et al., 2010), we hypothesized that disrupting HDAC3 activity within this cell-type would impair cocaine-induced locomotion. A higher dose of cocaine (10 mg/kg) was selected to observe any reductions in cocaine-induced locomotion. However, we found that disrupting HDAC3 activity within D2R-MSNs did not alter cocaine-induced locomotion (three-way ANOVA: main effect of cocaine: *F*_(4,4)_ = 265.7, *p* < 0.0001; no main effect of virus: *F*_(1,4)_ = 3.312, *p* = 0.0738; no main effect on session: *F*_(4,4)_ = 1.819, *p* = 0.1370; Fig. 7*E*). Altogether, this data suggests that HDAC3 does not regulate cocaine-induced locomotion within either D1R-MSNs or D2R-MSNs.

Given that anxiety can affect performance in the cocaine CPP task, we tested the effects of HDAC3-Y298H-v5 in D1R-MSNs versus D2R-MSNs (Pelloux et al., 2009) using the EPM. D1R-Cre male and female mice spent similar amount of time in open arms versus closed arms regardless of DIO-HDAC3-Y298H-v5 manipulation (two-way ANOVA RM: main effect of arm: *F*_(1,19)_ = 462.1, *p* < 0.0001; no main effect of virus: *F*_(1,19)_ = 0.97 806, *p* = 0.7830; no interaction: *F*_(1,19)_ = 0.007178, *p* = 0.9334; Fig. 6*G*,*H*). HDAC3-Y298H-v5 in D2R-MSNs had similarly not affected anxiety-like behaviors (two-way ANOVA RM: main effect of arm: *F*_(1,36)_ = 494.8, *p* < 0.0001; no main effect of virus: *F*_(1,36)_ = 0.2966, *p* = 0.5894; no interaction: *F*_(1,36)_ = 0.609, *p* = 0.4403; Fig. 7*F*,*G*). Collectively, the above data indicate that disrupting HDAC3's deacetylase activity within D1R-MSNs does not affect baseline anxiety, and its role may thus be specific to cocaine-associated behaviors.

To further examine whether D1R-specific HDAC3 activity regulates cocaine-induced behaviors, we tested the effects our D1R-HDAC3 manipulation on cocaine IVSA. Given our CPP data, we hypothesized that disrupting HDAC3 activity within D1-MSNs would enhance cocaine self-administration. D1R-HDAC3-Y298H-v5 mice and D1R-mcherry mice similar cocaine intake (two-way RM ANOVA: main effect on session: *F*_(9,171)_ = 11.04, *p* < 0.0001; no main effect on virus: *F*_(1,19)_ = 0.4572, *p* = 0.4572; no interaction: *F*_(9,171)_ = 0.2082, *p* = 0.9929; Fig. 6*J*) and learned to discriminate similarly between the active lever and inactive lever (D1R-mCherry: two-way RM ANOVA: main effect on session: *F*_(9,216)_ = 8.302, *p* < 0.0001; no main effect on lever: *F*_(1,24)_ = 63.44, *p* < 0.0001; interaction: *F*_(9,216)_ = 4.495, *p* < 0.0001; D1R-HDAC3-Y298H-v5: two-way RM ANOVA: main effect on session: *F*_(9,144)_ = 7.719, *p* < 0.0001; no main effect on lever: *F*_(1,16)_ = 51.95, *p* < 0.0001; no interaction: *F*_(9,144)_ = 1.527; Fig. 6*K*). Therefore, in contrast to our hypothesis, disrupting HDAC3 activity within D1R-MSNs does not affect cocaine intake; 24 h following the last cocaine IVSA, mice underwent a cocaine seeking test, to determine whether D1-HDAC3-Y298H-v5 affects abstinence-induced cocaine-seeking. D1R-HDAC3-Y298H-v5 significantly decreased cocaine seeking 24 h and 30 d after the last cocaine session (cocaine seeking test 1: unpaired *t* test *t*_(19)_ = 2.298, *p* < 0.05; cocaine seeking test 2: unpaired *t* test *t*_(19)_ = 2.274, *p < 0.05; Fig. 6*K*,*L*). Representative event records for D1R-HDAC3-Y298H-v5 and D1R-mcherry mice during cocaine seeking test day 1 are shown in Figure 6*M*. These data suggest that disrupting D1R-HDAC3 activity does not affect within session extinction learning, but instead persistently alters seeking following cocaine self-administration. This further demonstrates that HDAC3 activity within D1R MSNs regulates cocaine-induced behaviors.

## Discussion

Here, we show that HDAC3's deacetylase activity is a mediator of cocaine-induced plasticity within the NAc. More specifically, we illustrate how cocaine alters HDAC3 activity in a target-specific manner to promote changes in plasticity-related gene expression. In addition, cocaine-induced cellular processes were altered following disruption of HDAC3's deacetylase activity. We observed that chronic cocaine exposure induced cell-type-specific effects on *Hdac3* expression in D1R-MSNs but not D2R-MSNs. These D1R-MSN-specific changes in *Hdac3* expression may underlie cocaine-induced behaviors, as disrupting HDAC3's deacetylase activity within D1R-MSNs, but not D2R-MSNs, altered cocaine CPP and cocaine-seeking. These data suggest that HDAC3 has a cell-type-specific role in driving cocaine-induced processes within the NAc to regulate behavioral responses to cocaine.

Our molecular data suggest that HDAC3 activity is altered by chronic cocaine within the NAc to promote changes in gene expression of *Nr4a1* and *Fos*. These cocaine-induced changes in *Nr4a1* expression may be mediated by changes in H4K8Ac. HDAC3's deacetylase activity thus may be critical in driving downstream functions of Nr4a1, as Nr4a1 is critical in regulating both memory formation and cocaine action (Kwapis et al., 2019; Carpenter et al., 2020). Interestingly, although HDAC3 occupancy was reduced at the Fos promoter, no changes in H4K8Ac were detected. This may indicate that alternative promoter regions or histone marks are altered following the removal of HDAC3 at the Fos promoter. However, HDAC3 may not regulate expression of all Nr4a genes in the NAc following chronic cocaine. For instance, although we find that *Nr4a3* is induced following cocaine exposure (Guez-Barber et al., 2011; Hawk et al., 2012), our data suggest that these changes are not HDAC3-dependent. Our lack of changes in *Nr4a2* expression and HDAC3 activity observed at the Nr4a2 promoter following chronic cocaine could indicate that additional molecular mechanisms, HDAC3 dependent or independent, prevent *Nr4a2/3* expression following cocaine exposure within the NAc. Alternatively, *Nr4a2* is differentially expressed within the main cell-types of the NAc following chronic cocaine exposure (Chandra et al., 2015). Therefore, our methods of global tissue analysis are not sufficient to capture these cell-type-specific changes of *Nr4a2*. In our HDAC3-Y298H-v5 experiments, we show that *Nr4a2* expression is increased following HDAC3-Y298H-v5 overexpression. It should be noted that in our EV-Controls samples, cocaine decreases *Nr4a2* expression and this is restored by our HDAC3-Y298H-v5 manipulation. The variability in cocaine-induced profiling of *Nr4a2* expression between Figure 1*C–E* and *J* could be because our sample are from heterogeneous NAc cellular populations. Our findings still demonstrate disrupting HDAC3 activity is sufficient to alter *Nr4a2* expression following cocaine exposure. This supports the idea that HDAC3 regulates cocaine-induced changes at this time point. However, cell-type-specific studies would need to be conducted to determine whether HDAC3 negatively regulates *Nr4a2* primarily within one cell-type and whether this regulation is critical in driving cocaine-induced behaviors. Altogether, our data suggest that cocaine alters HDAC3 activity to promote plasticity-related gene expression, which is partially attributed to target-specific changes in histone acetylation.

Although the goal of this study was to understand HDAC3's response to cocaine and its role in cocaine-induced behaviors, it remains unknown what upstream signaling cascades recruit HDAC3 function. This may occur through cocaine-induced changes in the phosphorylation state of HDAC3 (Hervera et al., 2019), or HDAC3's interactions with NCOR1 or NCOR2, two proteins that have DNA-binding domains within the HDAC3 transcriptional repressor complex (Li et al., 2000; Sun et al., 2013). Although no changes in *Ncor1* or *Ncor2* expression were seen, follow-up studies may examine whether these interactions with HDAC3 have changed. It is also possible cocaine alters localization of HDAC3, similar to HDAC4 and HDAC5 (Taniguchi et al., 2012); however, HDAC3 is considered a mainly nuclear protein. Thus, although the mechanism is still unclear, our data do illustrate the importance of HDAC3's deacetylase activity in cocaine-induced expression in the NAc.

Although some studies report that chronic exposure of a pharmacological HDAC inhibitor further cocaine-induced depression of LTP, in contrast, we found that disrupting HDAC3 activity restored LTP in the NAc of cocaine-conditioned mice. These discrepancies may be because of differences in approaches (Levine et al., 2011) or distinct mechanisms underlying these effects (Finnin et al., 1999). Future studies parsing apart the different roles of each HDAC in cocaine-induced synaptic plasticity will be critical in better understanding the mechanisms underlying these results. It is unclear whether the restoration of LTP results from HDAC'3 deacetylase activity is accelerating processes of cocaine action, such as increases in spine density or unsilencing synapses within the NAc. Other studies have found similar effects on cocaine-related excitability when overexpressing the transcription factor CREB (Dong et al., 2006). Therefore, disruption of HDAC3 activity may promote CREB-dependent transcription and excitability. Overall, these findings shed light on possible mechanisms of action underlying changes in NAc cellular responses.

Our RNAScope data indicate that cocaine differentially alters expression of HDAC3 in D1R versus D2R-MSNs of the NAc. These changes may be a counteradaptive response, whereby *Hdac3* expression is increased because of repeated increases in transcriptional activity within D1R-MSNs (Chandra et al., 2015) and the decreased HDAC3 activity in the NAc, which we found from our ChIP-qPCR data. In D2R-MSNs, cocaine decreases expression of IEGs (Chandra et al., 2015), thus potentially this results in compensated decreases in repressors such as Hdac3. Additional studies determine whether this is time point specific or paradigm specific may further illustrate this mechanism.

Consistent with our D1R-MSN changes in *Hdac3* expression, our behavioral data indicate that HDAC3's deacetylase activity within D1R-MSNs, but not D2R-MSNs, regulates cocaine-induced behaviors. It is critical to note that our D1R-Cre control mice did not form cocaine-CPP at doses typically seen with this task (White et al., 2016; Alaghband et al., 2018; López et al., 2019b). Although animals were backcrossed at a minimum of seven generations to C57BL/6-J mice, this transgenic line may have underlying issues related to memory formation. However, our data show that regardless of dose or sex and across cohorts, disrupting HDAC3 activity is sufficient to enhance CPP in comparison to our control mice. This is consistent with other studies that have reported D1R-specific molecular mechanisms in the NAc that regulate behavior (Heshmati et al., 2018; Parekh et al., 2019). Within D2R-MSNs it is possible other functions or corepressor proteins play a more critical role in regulating cocaine-induced transcriptional changes. HDAC3's deacetylase activity may also create a permissive state for transcription to occur in both D1R-MSNs and D2R-MSNs, yet the signaling and activity that is required for transcription to occur is absent/reduced in D2R-MSNs following cocaine exposure (Calipari et al., 2016). Therefore, altered HDAC3 activity alone in D2R-MSNs may be insufficient to cause robust behavioral changes. Future studies examining HDAC3's activity within D1R-MSNs versus D2R-MSNs using cell-type-specific ChIP sequencing may provide more insight into the exact mechanism at play.

In contrast to our CPP data, HDAC3 within D1R-MSNs does not affect acquisition of cocaine reinforcement, but reduces cocaine seeking on a subsequent testing day. This effect is persistent, as HDAC3-Y298H-v5 mice have reduced cocaine-seeking following 30-d withdrawal. This is consistent with other studies examining HDAC activity in the NAc which have shown to regulate aspects of cocaine and cue-primed reinstatement and incubation of craving in rats, but not cocaine reinforcement (Taniguchi et al., 2017; Li et al., 2018). Moreover, our impaired cocaine seeking effects presents a possible upstream mechanism for work from the Heller lab, where CRISPR-mediated overexpression of an HDAC3 target, Nr4a1, similarly resulted in decreased cocaine-seeking following cocaine IVSA (Carpenter et al., 2020). It is possible that the contrasting effects of CPP versus cocaine seeking from our D1R-HDAC3 manipulation, may reflect differences in HDAC3-dependent mechanisms when cocaine is self-administered, as physiological differences in experimenter-administered and cocaine-self administration are reported (Larson et al., 2011; McCutcheon et al., 2011; Anderson et al., 2018). Alternatively, there may be circuit-specific regulation of behaviors that are contributing to the contrasting behavioral results. D1R-MSNs have two main projection sites, ventral pallidum (VP) and ventral mesencephalon (VM), which have differential roles in cocaine-induced behaviors (Pardo-Garcia et al., 2019). NAc afferents also induce diverse changes in synaptic plasticity within the NAc (MacAskill et al., 2014; Shen et al., 2014; Barrientos et al., 2018). Depending on the context, cocaine may activate different inputs onto D1R-MSNs that recruits or excludes HDAC3-dependent activity. Alternatively, our HDAC3 manipulations could be primarily affecting D1R-projecting VP or VM cells, which contributed to paradigm-specific changes. Given that this is the first set of studies examining D1R-HDAC3 activity within this paradigm, follow-up studies examining how HDAC3 activity changes within D1R-MSNs and affects related circuits will further illustrate the underlying mechanism.

In summary, we found that disrupting HDAC3's deacetylase activity promotes cocaine-induced changes in histone acetylation and gene expression. In addition, HDAC3's activity regulates mechanisms underlying cocaine-induced changes in synaptic plasticity in the NAc. HDAC3 has a cell-type-specific role in regulating behavior, as disrupting HDAC3's activity within the D1R, but not D2R-MSNs regulate cocaine-seeking behaviors. These findings further support HDAC3's role as a negative regulator of cocaine-processes and illustrates how its enzymatic function within D1R-MSNs plays a role in molecular mechanisms that regulate drug-seeking behaviors.
